# Consistent choice of landscape urbanization level across the annual cycle in a migratory waterbird species

**DOI:** 10.1038/s41598-020-80872-3

**Published:** 2021-01-12

**Authors:** Amelia Chyb, Jan Jedlikowski, Radosław Włodarczyk, Piotr Minias

**Affiliations:** 1 Department of Biodiversity Studies and Bioeducation, Faculty of Biology and Environmental Protection, University of Łódź, Banacha 1/3, 90-237 Łódź, Poland; 2 Faculty of Biology, Biological and Chemical Research Centre, University of Warsaw, Żwirki i Wigury 101, 02-089 Warsaw, Poland

**Keywords:** Behavioural ecology, Urban ecology

## Abstract

Rapid urbanization has a great impact on avian distribution, ecology, habitat selection, and behavior. Recent avian studies indicated that individuals remain consistent in their behavioral responses to human disturbance across short periods of time. However, there is still little information about keeping consistent behaviors in distinct locations across different stages of the annual cycle. In this study, we aimed to test for long-term consistency in habitat selection with respect to urbanization in a migratory waterbird species, the Eurasian coot *Fulica atra*. For this purpose, we individually marked ca. 300 coots from four populations that varied in urbanization level and tracked their habitat preferences during the non-breeding season. We found that individuals from urban breeding populations selected habitats with a higher share of artificial areas during the non-breeding season, when compared to non-urban individuals. Also, a comparison of non-breeding sites selected by birds from our study populations with random sites showed that urban birds selected sites with higher urbanization level than resulting from random availability. Finally, we found a seasonal variation in habitat preferences in coots—individuals from all study populations selected more urbanized areas as the non-breeding season progressed. The results indicate that birds are able to remain consistent in habitat preferences not only at a large geographical scale, but also across different seasons. Marked between-population variation in habitat selection across the annual cycle may reflect personality differences of coots from urban and non-urban populations, and it stays in line with the personality-matching habitat choice hypothesis.

## Introduction

Rapid urbanization is considered to be one of the main causes of environmental changes at the global scale, such as habitat loss^1^, biotic homogenization^2,3^ and species extinction^4^. Already in 2008, more than half of the human population lived in cities, and this ratio is predicted to rise to 70% by 2050^5^. Urban land-use and land-cover alterations cause significant ecological changes in climatic regimes, phenology, and resource availability to wild animals^6,7^. Urbanized landscape is characterized by the increased human-related disturbance, noise and light pollution, traffic^8^, and altered predatory pressure (reduced pressure from natural predators and increased pressure from novel predators, such as domestic animals or humans)^9–13^. Significant feature of urban-dwelling animal populations is elevated population density, often resulting in increased competition and aggression towards conspecifics^14^. These specific conditions contribute to the emergence of the intense selection pressure, which prevents many species from living and breeding in human-dominated landscapes, making them urban avoiders. At the same time, the constantly expanding urban areas become a novel colonization ground for a small, but steadily growing number of species, mostly birds and mammals^14^. These species can establish and maintain stable populations in the urban areas (urban adapters), while some of them even become strictly dependent on urban resources (urban exploiters)^4^.

Within the class of birds, many species undergo the process of rapid urbanization^15,16^. Urban and non-urban individuals often differ in a wide array of ecological, physiological, and genetic traits^8,17–19^, but behavioral divergence is probably most apparent^20^. First, many urban birds show marked reduction in anxiety responses and increased boldness, which is manifested, for example, by the shorter flight initiation distance (FID) in response to an approaching human^21–25^. Many studies have also shown an elevated level of aggression of urban-dwelling individuals (towards humans and conspecifics), expressed by an active nest defense of the nest or aggressive territory behaviors^22,26^. Other adaptations to urban life may include the usage of anthropogenic nesting structures^27^ and prolonged daily activity caused by artificial light at night^28^. Many studies also showed lower baseline corticosterone levels^29,30^, as well as an attenuated acute corticosterone stress response^8^ and lower H/L ratios (proxy of physiological stress)^31^ in urban individuals. These results suggest that urban-dwelling birds reduce their levels of stress via alterations in their behavior to avoid it or via a weaker stress response^22^. Finally, the process of urbanization also significantly affects reproductive ecology of bird populations, e.g. milder microclimate and shorter retention of snow and ice cover enable earlier initiation of the breeding season in the urban areas^6,32^. Specific climate conditions in urbanized areas may also attract birds from adjacent wildland during severe winter weather^33^. Recent studies showed that average winter temperatures in urbanized areas may be even up to 3 °C higher than in rural surroundings (so called ‘urban heat island effect’)^34^, which may enhance food availability and winter survival of birds.

Despite the identification of many behavioral and ecological differences between urban and rural populations of birds, the mechanisms underlying urbanization processes remain poorly recognized. One of the key questions in urban ecology is whether the adaptations to life in human-dominated landscapes proceed primarily via phenotypic plasticity or microevolutionary changes. It seems likely that plasticity plays a leading role at the early stages of urban colonization processes, while some of the plastic adaptations may then become genetically fixed over time^35,36^. On the other hand, some non-urban individuals may be genetically pre-adapted to urban life, and they are more likely to settle and successfully reproduce in urban landscapes than random non-urban individuals (so-called genotype sorting)^37^. Thus, genetic differences between urban and non-urban populations may become apparent immediately after colonization event, and this genetic divergence may be enhanced by genetic drift associated with the establishment of new, often small, urban populations (so-called founder effect^38^). Although urban ecology studies have been rapidly accumulating over recent decades, most research on birds was conducted on resident urban populations (e.g.^39–42^) or during the breeding season of migratory urban species (e.g.^43^). In contrast, information on the ecology of migratory urban birds at the wintering sites is almost lacking^44^. Surprisingly, we also have limited knowledge on how these birds choose wintering habitats and whether this choice in terms of urbanization level is plastic or rather consistent across the annual cycle. We are aware of only one previous study about the consistency in disturbance tolerance at different annual stages in a long-distance migratory bird, the common crane *Grus grus*^45^.

The aim of this study was to assess differences in the choice of non-breeding habitats by migratory birds breeding in areas with different urbanization level. For this purpose, we chose a common reed-nesting waterbird, the Eurasian coot *Fulica atra*, which usually migrates on relatively short distances within its European part of range^46^. To obtain information on the choice of non-breeding habitats we captured and marked ca. 300 adult coots from four breeding populations in central Poland that markedly differed in the urbanization level: old urban population (established in the first half of twentieth century in the urban center of Warszawa), new urban population (established at the beginning of twenty-first century in the urban center of Łódź), suburban population (semi-natural sites around the urban center of Łódź), and non-urban population (two complexes of fish ponds located in rural landscape). We hypothesized that coots remain consistent in the selection of habitats across their annual cycle—individuals from both urban populations were expected to prefer more urbanized areas during non-breeding period, while birds from the suburban and non-urban populations were expected to avoid them. We also hypothesized that birds from the old urban population (Warszawa) should choose more urbanized non-breeding habitats than birds from the new urban population (Łódź), as the adaptations to urban life in the latter population were more likely to be plastic rather than genetically fixed.

## Material and methods

### General field procedures and study populations

**Figure 1:**
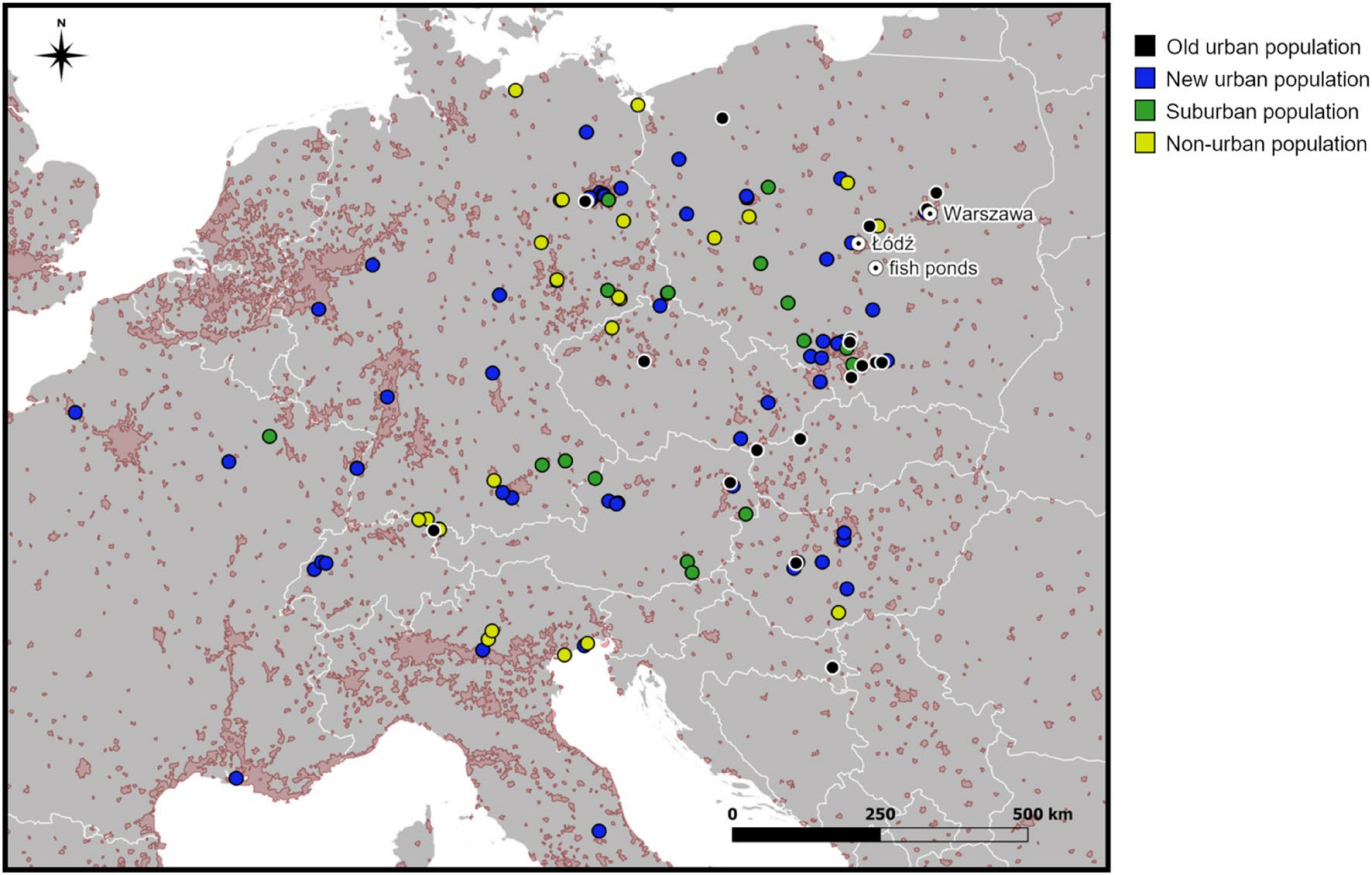
A map of non-breeding sites selected by Eurasian coots from different breeding populations in central Poland (old urban population—Warszawa; new and suburban populations—Łódź; non-urban population—fish ponds). A single non-breeding site (new urban population) in Spain was not shown because of outlying coordinates. The map was created in QGIS v. 3.16.0 (QGIS Development Team 2016, available at http://qgis.osgeo.org) and edited in GIMP v. 2.10.10 (GIMP Development Team 2019, available at: http://gimp.org ).

Eurasian coots were captured during ten breeding seasons (2010–2019) in four populations from Central Poland: two urban (old and new), one suburban, and one non-urban (Fig. 1). The old urban population was from Warszawa (52° 26′ N, 21° 02′ E), the largest city in Poland (1.77 million inhabitants; 517.24 km^2^). Breeding coots were reported from the center of Warszawa already in the middle of the twentieth century^47^, which makes it one of the first urban populations of coots established in the country. The second (new) urban population was from Łódź (51° 406′ N, 19° 28′ E), which also belongs to the largest cities in Poland (695 000 inhabitants; 293.25 km^2^). Coots colonized the center of Łódź in the 2000s, and the population steadily grew during the study period, reaching the final size of 30–40 pairs. However, semi-natural suburban sites around the center of Łódź (suburban population) were used by coots as breeding sites for a much longer time^48^. These suburban sites were characterized by a low share of the built-environment area and relatively low human disturbance, as well as semi-natural habitat structure (e.g., high availability of reed vegetation). Finally, birds from the non-urban population were captured at two nearby fish pond complexes: Sarnów (51° 51′ N, 19° 07′ E) and Żeromin (51° 37′ N, 19° 37′ E). These sites were located on the private properties with restricted trespassing for unauthorized personnel, resulting in low anthropogenic pressure.

In total, we captured 301 adult coots, although the sample sizes largely varied between populations. The highest number of coots was captured in the new urban population (Łódź), because of extensive long-term monitoring of this population (n = 158 adults). In contrast, sample sizes from other populations were much smaller (53 in the old urban populations, 35 adults in the suburban population, and 55 adults in the non-urban population). All birds were captured during incubation or while feeding on the shore with noose traps made from monofilament nylon or by hand (exclusively in urban populations). Each bird was marked with a metal ring (left tarsus) and plastic neck-collar with individual alphanumeric code, which allowed easy identification of birds in the field and greatly enhanced the resighting rate. At capture, we collected ca. 50 μl of blood from the ulnar vein into 96% ethanol, which was later used for molecular sexing. DNA was extracted using GeneJet Genomic DNA Purification Kit (Thermo Fisher Scientific, Waltham, MA, USA), and the sex-specific chromohelicase-DNA-binding gene was amplified using a protocol by Griffiths et al.^49^. All PCR products were separated on 2% agarose gel, and males were identified by one band only while females were identified by two bands. Bird capturing and blood sampling was performed by the permissions of the Local Bioethical Commission for Experiments on Animals in Łodź (nos 40/ŁB 620/2012 and 15/ŁB/2016) and complied with current laws of Poland.

Data on resightings were obtained from the database of the Polish Ringing Centre (PRC; http://ring.stornit.gda.pl), which collects data on resightings and recoveries of all birds ringed in Poland. The PRC compiles resighting information at both the national level, as well as it receives data from ringing centres in other countries. Usually, resighting information originates from both professional ornithologists and unprofessional bird-watchers, and it is strictly dependent on resighting effort. Nevertheless, all our resightings came from central and western European countries, where resighting effort should be relatively even and should not produce any major spatial bias in the resighting rate. In total, we obtained reliable information on the location of 148 non-breeding sites, which were defined as any site from outside the breeding season (July-March) located at least 20 km from the borders of the breeding population. All resightings were made at the water within 500 m from the shore or directly at the shore. Resightings of the same individual collected within a radius of 1 km within the same non-breeding season were treated as originating from a single site, and we used their mean coordinates to infer non-breeding habitat characteristics. In these cases, the date of the first resighting was used to determine when the site was occupied. In general, non-breeding season was divided into four periods of early autumn (July–September), late autumn (October–November), early winter (December-January), and late winter (February–March) and this categorization was used in the analyses. Resightings of the same individual at the same site in different non-breeding seasons were treated as separate data points, although we controlled for individual identity to avoid pseudoreplication. The number of data points per each breeding population roughly reflected the variation in our ringing effort, with 76 sites recorded for the new urban population and 20–29 sites recorded for the remaining three populations (Fig. 1).

### Habitat characteristics

To evaluate the habitat structure of sites chosen by coots in the non-breeding season, we measured a set of environmental variables around resighting locations. First, we used QGIS software (version 3.10.2, QGIS Development Team 2016) to create two different buffers of 500 m and 2.5 km around each point. The smaller buffer (500 m radius) was selected to reflect the home-rage size of wintering birds. To our knowledge, no data exist about the size of areas utilized by coots during the non-breeding season. Thus, we relied on one bird, which was observed 30 times at a single winter site (from December to February) and moved within a radius of ca. 600 m (unpublished data). The larger buffer (2.5 km radius) was selected to reflect habitat choice within a landscape scale. Subsequently, we acquired habitat information using the Corine Land Cover (CLC) layer from the 2018 year (geometric accuracy ≤ 10 m; minimum mapping unit/width 25 ha/100 m^50^). We determined the share of four habitat types within each buffer using level one of the CLC classification: (1) artificial areas (urban areas, transport units, mine, dump and construction sites; CLC classes 1.1–1.4); (2) agricultural areas (arable lands, crops, and pastures; CLC classes 2.1–2.4); (3) forest areas (forests, shrubs, and other natural or semi-natural areas with vegetation; CLC classes 3.1–3.3); (4) open water areas (all inland/marine waters and wetlands; CLC classes 4.1–4.2 and 5.1–5.2)^51^. Because artificial areas located around locations of coot non-breeding resightings were mainly urban fabric, urban parks and transport units (CLC classes 1.1, 1.2, 1.4) we used this trait as the primary index of urbanization level. Since agricultural lands are a dominant form of non-urban landscape in Central and Western Europe (main wintering grounds for our study populations), the share of agricultural areas was used as an additional index for the choice of non-urban habitats. All four habitat variables showed lack of significant correlations (artificial areas and open water areas; *P* > 0.15) or weak positive correlations (agricultural areas and forest areas; r < 0.3, *P* < 0.05) between both buffer scales.

To assess whether the choice of non-breeding sites by coots was selective with respect to habitat characteristics, the same procedure was also applied for a set of random points. We selected 100 random points within the main area occupied by coots during the non-breeding season, i.e. within the range of the observed longitudes and latitudes of non-breeding resightings (two outlier resightings were discarded as having their latitude/longitude < Q1 − 1.5 IQR or > Q3 + 1.5 IQR; Q1—first quartile, Q3—third quartile, IQR—interquartile range). All points were selected using QGIS random points tool, manually assigned to the nearest open water area based on 2018–2019 Landsat 8 satellite images^52^, and randomly located with the 500 m buffer from the shore (consistently with our resightings). We only considered water bodies with a minimum area of 5 ha or water-course with a minimum width of 30 m (minimal requirements for wintering coots from our populations, as inferred from our data). Finally, habitat variables were measured within the same buffers as around resighting locations.

### Statistical analyses

To assess differences in non-breeding habitat choice by coots from different breeding populations, we used general linear mixed models (GLMMs). Each habitat characteristic (artificial area, agricultural areas, forest areas, and open water areas) at each spatial scale (0.5 km and 2.5 km) was entered as a response variable in a separate model. Population, non-breeding period, and sex were entered as fixed factors, while longitude and latitude of non-breeding sites were entered as covariates to account for any possible geographical variation in habitat choice. We also included an interaction between population and sex to test for population-specific differences in habitat choice between males and females, but it was non-significant in all the models (*P* > 0.15) and removed. Since some individuals were recorded in multiple non-breeding locations or multiple times (in different non-breeding seasons) in the same non-breeding location, we have added individual identity as a random factor to avoid pseudoreplication resulting from repeated measurements of the same birds. The year was added as the second random factor to account for inter-annual variation in habitat choice. GLMMs with the same random factors were used to test for the differences in migratory distance and latitude/longitude of non-breeding resightings (response variables in separate models) between the populations. Sex and non-breeding period were entered as additional fixed factors in each of these models. Following recommendations by Nakagawa and Schielzeth^53^ we calculated fixed effects variance (marginal R^2^) and total variance explained (conditional R^2^) for each GLMM. For this purpose we used *r.squaredGLMM* function from *MuMIn* package^54^ developed for R statistical environment^55^. Goodness-of-fit was also estimated for each GLMM by comparing Akaike’s Information Criteria (AIC) for full and null models, as fitted using maximum likelihood approach. General linear models (GLMs) were used to test for the differences in habitat characteristics between non-breeding sites selected by coots from our study populations and random sites from the core wintering areas of these populations. All models were run in *lme4* R package^56^. All values are reported as means ± SE.

## Results

**Figure 2:**
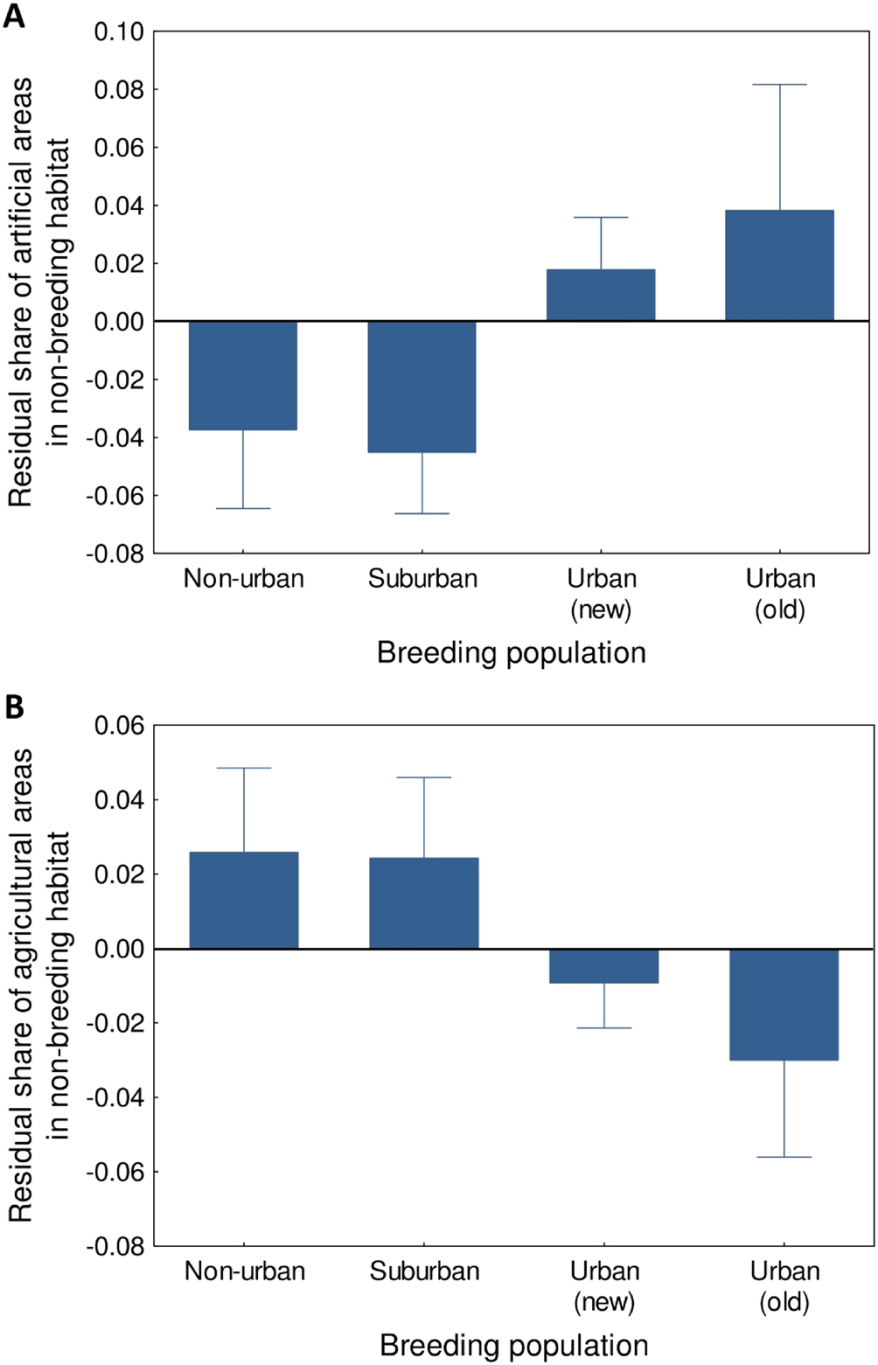
The share of artificial (**A**) and agricultural (**B**) areas in non-breeding habitats (0.5 km scale) selected by Eurasian coots from different breeding populations in central Poland. Residuals from general linear mixed models (means ± SE) are shown.

Our analysis provided evidence for differences in the choice of non-breeding habitats by coots from populations associated with different urbanization level. However, these differences were apparent exclusively at the small rather than large spatial scale. Most importantly, we found that non-breeding habitats differed significantly in the level of urbanization (0.5 km scale: W = 9.52, df = 3, *P* = 0.023), where birds from both urban breeding populations (Warszawa and Łódź) selected habitats with higher share of artificial areas during the non-breeding season, when compared to non-urban individuals (Warszawa: β = 0.185 ± 0.089, *P* = 0.037; Łódź: β = 0.101 ± 0.051, *P* = 0.048; Table 1; Fig. 2A). No significant differences in the share of artificial areas during the non-breeding season were found between coots from suburban and non-urban populations (Table 1; Fig. 2). We found significant differences in the share of agricultural areas in the non-breeding habitats selected by coots from different breeding populations (0.5 km scale: W = 8.35, df = 3, *P* = 0.039). Specifically, coots from the old urban population (Warszawa) selected non-breeding habitats with a lower share of agricultural areas when compared with coots from the non-urban population (β =  − 0.155 ± 0.067, *P* = 0.020; Table 1; Fig. 2B). There was a similar, but marginally non-significant association revealed for coots from the new urban population (Łódź: β =  − 0.072 ± 0.042, *P* = 0.086; Table 1; Fig. 2B). No significant differences in the share of agricultural areas during the non-breeding season were found between coots from suburban and non-urban populations (Table 1; Fig. 2). Also, no between-population variation was found in non-breeding habitat choice with respect to forest and open water areas (Tables S1–S2 in the Electronic Supplementary Material). Finally, no between-population variation in non-breeding habitat choice was recorded at the large (2.5 km) scale (Tables S3–S6).

**Table 1:** The results of general linear mixed models assessing variation in the share of artificial (A) and agricultural (B) areas in non-breeding habitats (0.5 km scale) selected by Eurasian coots from different breeding populations in central Poland. Year and individual identity were entered as random factors in each model. Marginal/conditional R^2^ for each model were 0.28/0.48 (A) and 0.15/0.44 (B), while ΔAIC was 26.0 (A) and 3.4 (B). Significant predictors are marked in bold.

Predictors	Estimate	Lower 95%CI	Upper 95%CI	*P*
**A. Artificial areas**				
**Intercept**	− **1.286**	− **2.017**	− **0.555**	**0.001**
Population (suburban vs. non-urban)	− 0.015	− 0.154	0.123	0.84
**Population** **(new urban vs. non-urban)**	**0.101**	**0.001**	**0.201**	**0.048**
**Population** **(old urban vs. non-urban)**	**0.185**	**0.011**	**0.358**	**0.037**
**Non-breeding period** **(late autumn vs. early autumn)**	**0.144**	**0.044**	**0.243**	**0.005**
**Non-breeding period** **(early winter vs. early autumn)**	**0.202**	**0.106**	**0.298**	** < 0.001**
**Non-breeding period** **(late winter vs. early autumn)**	**0.223**	**0.121**	**0.325**	** < 0.001**
Sex (males vs. females)	− 0.016	− 0.099	0.067	0.72
Sex (undetermined vs. females)	− 0.016	− 0.193	0.162	0.87
Longitude	− 0.007	− 0.017	0.003	0.16
**Latitude**	**0.028**	**0.013**	**0.043**	** < 0.001**
**B. Agricultural areas**				
Intercept	0.472	− 0.083	1.026	0.095
Population (suburban vs. non-urban)	− 0.010	− 0.120	0.100	0.86
Population (new urban vs. non-urban)	− 0.072	− 0.155	0.010	0.086
**Population** **(old urban vs. non-urban)**	− **0.155**	− **0.286**	− **0.024**	**0.020**
Non-breeding period (late autumn vs. early autumn)	− 0.017	− 0.093	0.060	0.68
Non-breeding period (early winter vs. early autumn)	− 0.030	− 0.101	0.042	0.42
Non-breeding period (late winter vs. early autumn)	0.007	− 0.071	0.086	0.86
Sex (males vs. females)	0.016	− 0.047	0.079	0.63
Sex (undetermined vs. females)	0.086	− 0.049	0.222	0.21
**Longitude**	**0.012**	**0.005**	**0.020**	**0.001**
Latitude	− 0.010	− 0.021	0.002	0.10

A comparison of non-breeding sites selected by coots from our study populations with random sites showed that urban birds selected sites with higher share of artificial areas (Warszawa: β = 0.240 ± 0.050, *P* < 0.001; Łódź: β = 0.167 ± 0.031, *P* < 0.001) and lower share of agricultural areas (Warszawa: β =  − 0.117 ± 0.049, *P* = 0.018; Łódź: β =  − 0.145 ± 0.030, *P* < 0.001) than resulting from random availability. Non-breeding habitat choice (in terms of artificial and agricultural areas) by suburban and non-urban birds was consistent with random availability (all *P* > 0.05).

**Figure 3:**
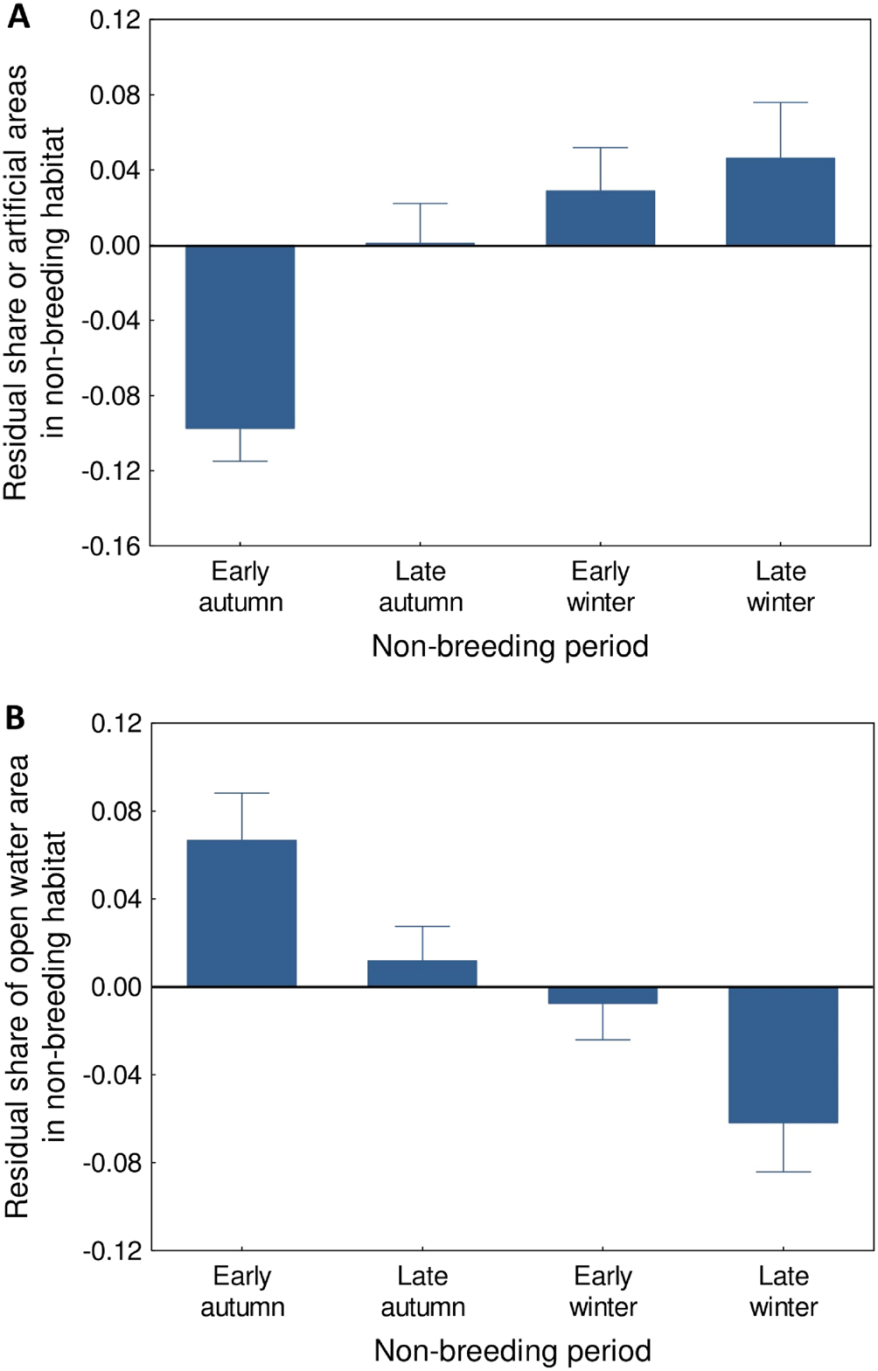
The share of artificial (**A**) and open water (**B**) areas in habitats (0.5 km scale) selected by Eurasian coots in different periods of non-breeding season. Residuals from general linear mixed models (means ± SE) are shown.

There was a significant seasonal variation in habitat selection by coots. The strongest variation during the non-breeding season was recorded for the urbanization level (0.5 km scale: W = 22.14, df = 3, *P* < 0.001; 2.5 km scale: W = 19.15, df = 3, *P* < 0.001), as we recorded a gradual transition towards habitats with higher share of artificial areas as the season progressed (Table 1; Fig. 3A). Coots were observed in the most urbanized habitats in early and late winter, and the share of urbanized landscape in these periods was significantly higher than in early autumn (Table 1; Fig. 3A). This pattern was consistent across all our study breeding populations, as found by non-significant population vs. non-breeding period interactions (0.5 km scale: W = 5.20, df = 8, *P* = 0.74; 2.5 km scale: W = 6.95, df = 8, *P* = 0.54). There was also a gradual transition towards a lower share of open water in the habitats during the winter period, but it was apparent only at the small spatial scale (early winter vs. early autumn: β =  − 0.150 ± 0.047, *P* = 0.001; late winter vs. early autumn: β =  − 0.226 ± 0.048, *P* < 0.001; Table 1; Fig. 3B). No differences in non-breeding habitat choice were found between sexes (Table 1, S2–S4, S6), except for males choosing significantly (2.5 km scale) or marginally non-significantly (0.5 km scale) more forested areas than females (Table S1, S5).

We have recorded no significant variation in migratory distance and mean latitude/longitude of non-breeding resightings between the populations (all *P* > 0.05), indicating that differences in non-breeding habitat choice were not driven by different migratoriness or geographical separation of wintering grounds of urban and non-urban coots. Also, there was no between-sex variation in migratory distance and mean coordinates of non-breeding resightings (all *P* > 0.05). In contrast, we found significant differences in migratory distance between consecutive stages of non-breeding season (W = 11.35, *P* = 0.010), with a significant increase in migratory distance between early and late autumn (323.1 ± 59.0 km vs. 480.2 ± 60.8 km; *P* = 0.009) and longest migratory distance recorded during late winter (516.0 ± 58.7 km; *P* = 0.002 compared to early autumn). This seasonal increase in migratory distance was primarily driven by latitudinal movements of coots (W = 9.92, *P* = 0.019), as we recorded a significant decrease in the mean latitude of resightings between early autumn and late winter (50.80 ± 0.48° N vs. 49.63 ± 0.48° N; *P* = 0.009). At the same time, we recorded no significant differences in the mean longitude of resightings between consecutive stages of non-breeding period (W = 5.72, *P* = 0.13).

## Discussion

In this study, we provide empirical support for consistency in habitat selection across the annual cycle of a migratory waterbird species, the Eurasian coot. We found that coots from urban breeding populations (Warszawa and Łódź) selected habitats with a higher share of artificial areas during the non-breeding season when compared to non-urban individuals. These differences in non-breeding habitat choice could not be attributed to variation in migratoriness or geographical separation of wintering grounds, as urban and non-urban coots showed similar migratory distances and did not differ in the mean latitude and longitude of non-breeding resightings. A comparison of non-breeding sites selected by birds from our study populations with random sites showed that urban birds selected sites with a higher share of artificial areas and lower share of agricultural areas than resulting from random availability. Finally, we found support for a seasonal variation in habitats selection by coots—birds from all study populations were observed in more urbanized habitats in winter than in early autumn.

Recent studies provide a strong body of evidence that birds remain consistent in their behavioral responses to different stimuli across short periods of time^41,57,58^, but there is still little information about keeping consistent behaviors in distinct locations across the entire annual cycle. A study by Vegvari et al.^45^ was one of the notable exceptions, showing that common cranes remained consistent in the tolerance of human disturbance at a large geographical scale, i.e. between natal and wintering sites, which are not only separated by large distances, but also differ ecologically. In this study four of the five components of disturbance tolerance were also highly repeatable in time (within and between years) for individual cranes at the migratory stop-over site^45^.

Consistent disturbance tolerance behavior of migratory species may be a result of three mutually non-exclusive mechanisms. First, consistent individual differences in habitat selection may be the effect of an adaptation to a certain level of human disturbance and landscape urbanization. Birds may select habitats across the entire annual cycle based on local conditions, to which they are best adapted in terms of their morphology, physiology, and behavior (‘matching habitat choice’^59^). Second, habitat preferences may be heritable to a certain extent and, thus, consistent habitat selection across space and time may be reinforced at the genetic level^60^. Third, the choice of similar habitats (e.g., with a similar level of anthropogenic disturbance) during different phases of the annual cycle may be a result of early natal experiences (‘natal habitat preference induction’—NHPI^61^). Our study indicated that coots from both old and new urban breeding populations showed a marked tendency to select habitats with a higher share of artificial areas during the non-breeding season when compared to their non-urban conspecifics. The presence of such relationship in the new urban population may suggest that consistency in habitat selection across the entire annual cycle is the result of behavioral plasticity or NHPI rather than the genetic background. The analysis of microsatellite variation indicated that coot population from Łódź was genetically more similar to neighboring semi- and non-urban populations than to old urban populations from Poland^62^. Łódź was colonized quite recently (beginning of twenty-first century) probably through an influx of individuals from the surrounding wildland (model of independent urban colonization) and microevolutionary adaptations related to a novel urban environment are unlikely to be fixed at the genetic level in such a short period of time since the colonization^62^.

Temporally consistent between-individual variation in response to different stimuli may lead to a non-random distribution of behavioral phenotypes across available habitats. An increasing body of evidence indicates that animals may select habitats that best suit their personality (personality-matching habitat choice hypothesis^63^). In the class of birds, the personality-dependent distribution of individuals across heterogeneous environment was confirmed by studies of behavioral traits such as aggressiveness and risk-taking behavior^63,64^. Our earlier research indicated that urban breeding coots also present a wide range of behavioral adaptations to increased human disturbance in comparison to individuals from non-urban population, including elevated boldness and reduced fear of humans^31^. Behavior of coots from the newly-established urban population was consistent with behavioral syndromes found in typical urban exploiters (a part of a general ‘aggression syndrome’ of urban wildlife^22^). Thus, we suggest that marked personality differences in behavior of coots from urban and non-urban populations may lead to consistency in habitat selection across the annual cycle (in line with the personality-matching habitat choice hypothesis).

Urban areas play an important role as wintering sites for sedentary species of birds^39,44,65^. Our study shows that urban areas may also act as important wintering sites for migratory waterbirds, as we recorded a gradual transition towards habitats with a higher share of artificial areas as the non-breeding season progressed, and this pattern was consistent across all study populations. It is probably the effect of different climatic conditions and different availability of food resources in habitats with varying level of urbanization. Human-dominated landscape usually has lower availability of natural food resources (as the effect of reduced natural land cover), so it could be a less beneficial habitat for waterbirds in autumn when natural waters are still uncovered with ice. In contrast, milder microclimate (heat islands) and supplementary feeding by humans make the urban environment more favorable for coots and other waterbirds during the winter season^7,66,67^.

During the non-breeding season, several species of territorial migratory birds exhibit a non-random pattern of habitat distribution, with some areas occupied predominately by males and others predominately by females. Sexual habitat segregation is considered to be the effect of intraspecific competition for limited resources^68–70^ or innate preferences of males and females to different types of habitat^71^. We have found weak evidence for an occurrence of sexual habitat segregation in coots (males tended to prefer more forested areas). However, no significant differences in non-breeding habitat choice were found between sexes in terms of urbanization level. Weak sexual segregation in space may be the effect of relatively low intraspecific competition for food during the non-breeding season, as the Eurasian coot is highly sociable species outside the reproductive period^72^.

In conclusion, our study indicated that Eurasian coots remain consistent in the choice of habitat urbanization level across their annual cycle. Non-random spatial distribution of birds across different phases of the annual cycle may be the reason of limited behavioral plasticity, as ecological conditions in similar habitats are expected to remain relatively stable in time. Also, consistent segregation with respect to habitat urbanization may facilitate faster genetic divergence between urban and non-urban population and genetic fixation of urban-related adaptations. Although our research indicated the unequivocal tendency of coots for a consistent habitat selection in terms of urbanization level across the annual cycle, the exact behavioral or genetic mechanisms underlying this phenomenon should be further investigated, preferably in the sampling framework based on multiple replicates of urban and non-urban populations.

## Supplementary Information

Supplementary information 1.

Supplementary information 2.

Supplementary Tables.

## Data Availability

Raw data and R code are available as Electronic Supplementary Material.
