# Supplementary material for: Consistent choice of landscape urbanization level across the annual cycle in a migratory waterbird species
Source: Sci Rep. 2021 Jan 12;11:836. doi: 10.1038/s41598-020-80872-3 (PMC7804327; doi:10.1038/s41598-020-80872-3)
Supplement: Supplementary file 1 — Supplementary information 1. [file 41598_2020_80872_MOESM1_ESM.docx]

**Supplementary R code**

**Consistent choice of landscape urbanization level across the annual cycle in a migratory waterbird species**

**Amelia Chyb^1^, Jan Jedlikowski^2^, Radosław Włodarczyk^1^, Piotr Minias^1^**

^1^ Department of Biodiversity Studies and Bioeducation, Faculty of Biology and Environmental Protection, University of Łódź, Banacha 1/3, 90-237 Łódź, Poland

^2^ Faculty of Biology, Biological and Chemical Research Centre, University of Warsaw, Żwirki i Wigury 101, 02-089 Warsaw, Poland

Correspondence and requests for materials should be addressed to P.M. (email: pminias@op.pl)

# General Linear Mixed Models for non-breeding habitat choice, migratory distance and longitude/latitude

library(lme4)

GLMM_habitat <- lmer (Habitat_trait ~ Population + Non-breeding_period + Sex + Longitude + Latitude + (1|Year) + (1|Bird_ID), data = mydata)

GLMM_migratory_distance <- lmer (Migratory_distance ~ Population + Non-breeding_period + Sex + (1|Year) + (1|Bird_ID), data = mydata)

GLMM_coordinates <- lmer (Longitude/latitude ~ Population + Non-breeding_period + Sex + (1|Year) + (1|Bird_ID), data = mydata)

# Estimating marginal and conditional R^2^

library(MuMIn)

R2m<-r.squaredGLMM(GLMM_habitat)[1]

R2c<-r.squaredGLMM(GLMM_habitat)[2]

# Estimating AIC

GLMM_habitat_full <- lmer (Habitat_trait ~ Population + Non-breeding_period + Sex + Longitude + Latitude + (1|Year) + (1|Bird_ID), data = mydata, REML = FALSE)

GLMM_habitat_null <- lmer (Habitat_trait ~ 1 + (1|Year) + (1|Bird_ID), data = mydata, REML = FALSE)
