# Supplementary material for: Consistent choice of landscape urbanization level across the annual cycle in a migratory waterbird species
Source: Sci Rep. 2021 Jan 12;11:836. doi: 10.1038/s41598-020-80872-3 (PMC7804327; doi:10.1038/s41598-020-80872-3)
Supplement: Supplementary file 3 — Supplementary Tables. [file 41598_2020_80872_MOESM3_ESM.doc]

**Electronic Supplementary Material**

**Consistent choice of landscape urbanization level across the annual cycle in a migratory waterbird species**

**Amelia Chyb^1^, Jan Jedlikowski^2^, Radosław Włodarczyk^1^, Piotr Minias^1^**

^1^ Department of Biodiversity Studies and Bioeducation, Faculty of Biology and Environmental Protection, University of Łódź, Banacha 1/3, 90-237 Łódź, Poland

^2^ Faculty of Biology, Biological and Chemical Research Centre, University of Warsaw, Żwirki i Wigury 101, 02-089 Warsaw, Poland

Correspondence and requests for materials should be addressed to P.M. (email: pminias@op.pl)

**Table S1** The results of the general linear mixed model assessing variation in the share of forest areas in non-breeding habitats (0.5 km scale) selected by Eurasian coots from different breeding populations in central Poland. A year and individual identity were entered as random factors in each model. Marginal / conditional R^2^ were 0.08 / 0.35, while ΔAIC = -9.2.

| Predictors | Estimate | Lower 95%CI | Upper 95%CI | P |
| --- | --- | --- | --- | --- |
| Intercept | -0.092 | -0.378 | 0.195 | 0.54 |
| Population  (suburban vs. non-urban) | -0.038 | -0.092 | 0.017 | 0.18 |
| Population  (new urban vs. non-urban) | -0.026 | -0.065 | 0.013 | 0.19 |
| Population  (old urban vs. non-urban) | -0.065 | -0.133 | 0.004 | 0.063 |
| Non-breeding period  (late autumn vs. early autumn) | -0.021 | -0.059 | 0.018 | 0.29 |
| Non-breeding period  (early winter vs. early autumn) | 0.003 | -0.035 | 0.040 | 0.90 |
| Non-breeding period  (late winter vs. early autumn) | -0.003 | -0.043 | 0.036 | 0.88 |
| Sex (males vs. females) | 0.022 | -0.010 | 0.055 | 0.18 |
| Sex (undetermined vs. females) | 0.019 | -0.051 | 0.089 | 0.62 |
| Longitude | -0.001 | -0.005 | 0.002 | 0.47 |
| Latitude | 0.003 | -0.002 | 0.009 | 0.25 |

**Table S2** The results of the general linear mixed model assessing variation in the share of open water areas in non-breeding habitats (0.5 km scale) selected by Eurasian coots from different breeding populations in central Poland. A year and individual identity were entered as random factors in each model. Marginal / conditional R^2^ were 0.18 / 0.62, while ΔAIC = 12.6. Significant predictors are marked in bold.

| Predictors | Estimate | Lower 95%CI | Upper 95%CI | P |
| --- | --- | --- | --- | --- |
| **Intercept** | **1.619** | **0.866** | **2.373** | **<0.001** |
| Population  (suburban vs. non-urban) | 0.054 | -0.101 | 0.210 | 0.50 |
| Population  (new urban vs. non-urban) | 0.005 | -0.103 | 0.112 | 0.94 |
| Population  (old urban vs. non-urban) | 0.008 | -0.177 | 0.193 | 0.94 |
| Non-breeding period  (late autumn vs. early autumn) | -0.084 | -0.176 | 0.008 | 0.072 |
| **Non-breeding period**  **(early winter vs. early autumn)** | **-0.150** | **-0.243** | **-0.058** | **0.001** |
| **Non-breeding period**  **(late winter vs. early autumn)** | **-0.226** | **-0.320** | **-0.131** | **<0.001** |
| Sex (males vs. females) | -0.024 | -0.115 | 0.067 | 0.62 |
| Sex (undetermined vs. females) | -0.092 | -0.287 | 0.104 | 0.36 |
| Longitude | -0.003 | -0.013 | 0.008 | 0.64 |
| **Latitude** | **-0.021** | **-0.037** | **-0.006** | **0.007** |

**Table S3** The results of the general linear mixed model assessing variation in the share of artificial areas in non-breeding habitats (2.5 km scale) selected by Eurasian coots from different breeding populations in central Poland. A year and individual identity were entered as random factors in each model. Marginal / conditional R^2^ were 0.23 / 0.50, while ΔAIC = 17.3. Significant predictors are marked in bold.

| Predictors | Estimate | Lower 95%CI | Upper 95%CI | P |
| --- | --- | --- | --- | --- |
| **Intercept** | **-26.556** | **-44.052** | **-9.060** | **0.003** |
| Population  (suburban vs. non-urban) | -0.266 | -3.654 | 3.122 | 0.89 |
| Population  (new urban vs. non-urban) | 2.065 | -0.339 | 4.470 | 0.092 |
| Population  (old urban vs. non-urban) | 2.240 | -1.953 | 6.434 | 0.30 |
| **Non-breeding period**  **(late autumn vs. early autumn)** | **3.666** | **1.362** | **5.970** | **0.002** |
| **Non-breeding period**  **(early winter vs. early autumn)** | **4.552** | **2.295** | **6.808** | **<0.001** |
| **Non-breeding period**  **(late winter vs. early autumn)** | **4.536** | **2.170** | **6.902** | **<0.001** |
| Sex (males vs. females) | -0.567 | -2.593 | 1.460 | 0.60 |
| Sex (undetermined vs. females) | 2.279 | -2.057 | 6.615 | 0.31 |
| Longitude | -0.051 | -0.283 | 0.182 | 0.68 |
| **Latitude** | **0.574** | **0.216** | **0.932** | **0.002** |

**Table S4** The results of the general linear mixed model assessing variation in the share of agricultural areas in non-breeding habitats (2.5 km scale) selected by Eurasian coots from different breeding populations in central Poland. A year and individual identity were entered as random factors in each model. Marginal / conditional R^2^ were 0.21 / 0.62, while ΔAIC = 10.9. Significant predictors are marked in bold.

| Predictors | Estimate | Lower 95%CI | Upper 95%CI | P |
| --- | --- | --- | --- | --- |
| Intercept | 11.864 | -2.868 | 26.596 | 0.11 |
| Population  (suburban vs. non-urban) | 2.566 | -0.516 | 5.649 | 0.10 |
| Population  (new urban vs. non-urban) | -0.446 | -2.680 | 1.789 | 0.71 |
| Population  (old urban vs. non-urban) | -0.354 | -3.892 | 3.185 | 0.86 |
| Non-breeding period  (late autumn vs. early autumn) | -1.457 | -3.340 | 0.426 | 0.13 |
| **Non-breeding period**  **(early winter vs. early autumn)** | **-2.403** | **-4.207** | **-0.598** | **0.009** |
| Non-breeding period  (late winter vs. early autumn) | -1.425 | -3.352 | 0.501 | 0.15 |
| Sex (males vs. females) | -1.002 | -2.740 | 0.736 | 0.26 |
| Sex (undetermined vs. females) | -2.126 | -5.876 | 1.623 | 0.27 |
| **Longitude** | **0.370** | **0.174** | **0.565** | **<0.001** |
| Latitude | -0.191 | -0.494 | 0.112 | 0.22 |

**Table S5** The results of the general linear mixed model assessing variation in the share of forest areas in non-breeding habitats (2.5 km scale) selected by Eurasian coots from different breeding populations in central Poland. A year and individual identity were entered as random factors in each model. Marginal / conditional R^2^ were 0.11 / 0.33, while ΔAIC = -3.9. Significant predictors are marked in bold.

| Predictors | Estimate | Lower 95%CI | Upper 95%CI | P |
| --- | --- | --- | --- | --- |
| Intercept | 1.381 | -9.145 | 11.907 | 0.81 |
| Population  (suburban vs. non-urban) | -0.621 | -2.598 | 1.356 | 0.55 |
| Population  (new urban vs. non-urban) | 0.024 | -1.410 | 1.458 | 0.98 |
| Population  (old urban vs. non-urban) | -2.193 | -4.689 | 0.304 | 0.085 |
| Non-breeding period  (late autumn vs. early autumn) | -1.296 | -2.735 | 0.143 | 0.077 |
| Non-breeding period  (early winter vs. early autumn) | -0.868 | -2.254 | 0.518 | 0.22 |
| Non-breeding period  (late winter vs. early autumn) | -1.460 | -2.934 | 0.015 | 0.052 |
| **Sex (males vs. females)** | **1.285** | **0.097** | **2.474** | **0.034** |
| Sex (undetermined vs. females) | 1.396 | -1.146 | 3.939 | 0.29 |
| Longitude | -0.079 | -0.218 | 0.059 | 0.26 |
| Latitude | 0.073 | -0.142 | 0.287 | 0.52 |

**Table S6** The results of the general linear mixed model assessing variation in the share of open water areas in non-breeding habitats (2.5 km scale) selected by Eurasian coots from different breeding populations in central Poland. A year and individual identity were entered as random factors in each model. Marginal / conditional R^2^ were 0.21 / 0.67, while ΔAIC = 17.0. Significant predictors are marked in bold.

| Predictors | Estimate | Lower 95%CI | Upper 95%CI | P |
| --- | --- | --- | --- | --- |
| **Intercept** | **32.988** | **20.173** | **45.804** | **<0.001** |
| Population  (suburban vs. non-urban) | -1.358 | -4.039 | 1.323 | 0.33 |
| Population  (new urban vs. non-urban) | -1.103 | -2.952 | 0.746 | 0.25 |
| Population  (old urban vs. non-urban) | 0.046 | -3.123 | 3.215 | 0.98 |
| Non-breeding period  (late autumn vs. early autumn) | -0.531 | -2.053 | 0.992 | 0.50 |
| Non-breeding period  (early winter vs. early autumn) | -0.768 | -2.311 | 0.775 | 0.34 |
| Non-breeding period  (late winter vs. early autumn) | -1.513 | -3.084 | 0.058 | 0.059 |
| Sex (males vs. females) | 0.213 | -1.360 | 1.786 | 0.80 |
| Sex (undetermined vs. females) | -1.487 | -4.855 | 1.880 | 0.39 |
| **Longitude** | **-0.247** | **-0.421** | **-0.072** | **0.006** |
| **Latitude** | **-0.473** | **-0.737** | **-0.208** | **<0.001** |
